# Biomimetic Thermal-sensitive Multi-transform Actuator

**DOI:** 10.1038/s41598-019-44394-x

**Published:** 2019-05-27

**Authors:** Tae Hyeob Kim, Jung Gi Choi, Ju Young Byun, Yongwoo Jang, Sung Min Kim, Geoffrey. M. Spinks, Seon Jeong Kim

**Affiliations:** 1 Center for Self-Powered Actuation, Department of Biomedical Engineering, Hanyang University, Seoul, 04763 South Korea; 2 Department of Physical Education, Department of Active Aging Industry, Hanyang University, Seoul, 04763 South Korea; 3 ARC Centre of Excellence for Electromaterials Science, Intelligent Polymer Research Institute, University of Wollongong, Wollongong, New South Wales 2522 Australia

**Keywords:** Actuators, Polymers

## Abstract

Controllable and miniaturised mechanical actuation is one of the main challenges facing various emerging technologies, such as soft robotics, drug delivery systems, and microfluidics. Here we introduce a simple method for constructing actuating devices with programmable complex motions. Thermally responsive hydrogels based on poly(*N*-isopropylacrylamide) (PNIPAM) and its functionalized derivatives (f-PNIPAM) were used to control the lower critical solution temperature (LCST) or the temperature at which the gel volume changes. Techniques for ultra-violet crosslinking the monomer solutions were developed to generate gel sheets with controllable crosslink density gradients that allowed bending actuation to specified curvatures by heating through the LCST. Simple molding processes were then used to construct multi-transform devices with complex shape changes, including a bioinspired artificial flower that shows blossoming and reverse blossoming with a change in temperature.

## Introduction

The development of stimuli-responsive actuating materials is a promising solution to the need for controllable miniature mechanical motors in new technologies such as soft robotics^1–4^, drug delivery^5^, microfluidics^6^ and artificial muscles^7,8^. In particular, thermoresponsive hydrogel actuators have great potential in these fields because they can generate very large volume and shape changes and can be stimulated with various sources such as electrical Joule heating^9,10^, photothermal^11–14^, and exothermic reactions^15^. PNIPAM is the most widely used thermoresponsive hydrogel^16–22^ with a LCST around 36 °C. Combining PNIPAM-based hydrogels into a bilayer structure with a flexible passive material gives bending type actuators and recent reports demonstrate very large bending strokes^11,17^. By controlling the swelling anisotropy in the hydrogel layer, for example by preparing mats of aligned electrospun hydrogel layers^23,24^, it is also possible to fabricate bending, twisting, coiling and rolling shape changes.

A limitation of thermoresponsive hydrogels is their binary ‘on-off’ operation since the complete volume transition occurs over a narrow temperature range. Controlling the gel volume at intermediate states is difficult. One solution is to incorporate multiple actuator materials to give a multi-transforming structure where each actuator material responds to a different stimulus^17^. Patterning of the actuating elements within the device structure can produce complex shape transformations through a series of discrete manipulations^17,20^. To date, these demonstrated multi-transforming gel actuated structures need complicated fabrication methods such as electrospinning with controlled fiber orientation^23,25^ and stripe polymerization using photo-masking techniques^19,20,23,24^. In the present study, we demonstrate control of the gel actuation by incorporating variants of PNIPAM to give a range of LCSTs within the same device. Bending actuators are generated using an UV-photopolymerization method to control the crosslink density of the gel through the film thickness and simple molding methods are used to control the location of two different gel actuators within a single device. Using these methods we are able to generate multiple shapes through thermal control.

The LCST of NIPAM can be shifted by copolymerization with hydrophilic or hydrophobic materials. The LCST is shifted to a lower temperature by copolymerization with hydrophobic monomers and shifted to a higher temperature with hydrophilic monomers^26^. The f-PNIPAM copolymers used in the present study were based on monomer mixtures of NIPAM, acrylamide (AM) and 2,2′-hydroxyethyl methacrylate (HEMA). PNIPAM has its LCST around 36 °C. HEMA is material which makes LCST shift to lower temperature by polymerization with NIPAM. Acrylamide was used to shift LCST to higher temperature when it polymerized with NIPAM (Fig. S1). The ratio of these three monomers determines the LCST^26^. Controlling the monomer ratios and by using a simple UV-curing method gave hydrogels with two distinct temperature phase-transition points (48.0 °C and 60.8 °C).

## Results

### Multi-transforming flower

**Figure 1:**
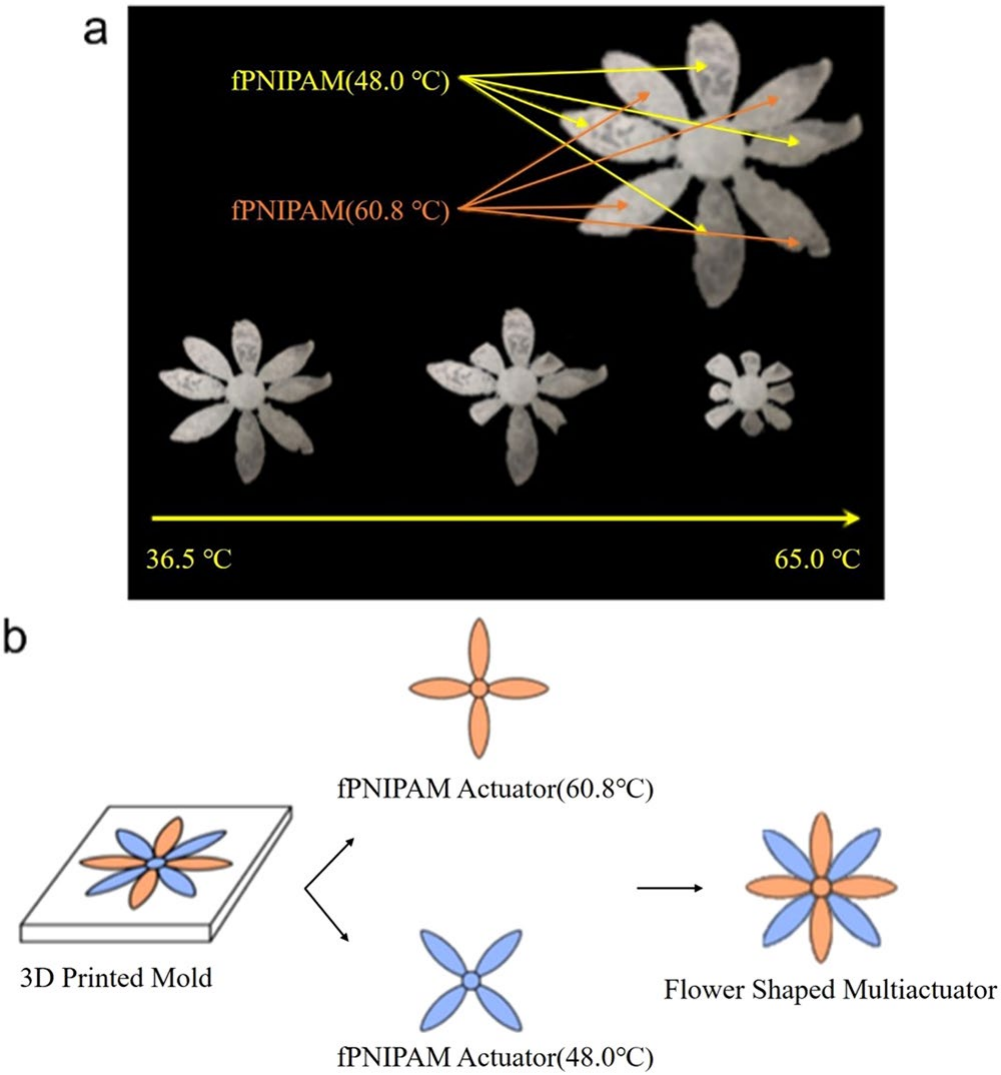
Flower blossom-shaped multi-transformation actuators. (**a**) Real-time image showing the different temperature-responsive actuation. The thickness of each actuator *t* = 2mm. The actuation occurs sequentially when the temperature rises. (**b**) Schematic image of the making process for the whole flower-type actuator structure. The two f-PNIPAM actuators, which have different LCSTs, were attached by *N*,*N*-bisacrylamide cross-linker.

A flower-shaped device illustrates the multi-transforming actuators that could be fabricated with f-PNIPAM materials (Fig. 1a). The f-PNIPAM flower shows reversible blossoming in two distinct phases as the temperature rises. At temperatures below the low LCST (48.0 °C), the artificial flower fully blossomed with its petals completely unfolded. The first transformation started near 48.0 °C by folding the four petals of the layer made from the hydrogel with the lower LCST. The other four petals that were made from the high LCST solution began to bend when the temperature rose to nearly 60.8 °C. For temperatures above the higher LCST, the device had completed its transformation and the flower shape resembled a bud. The device needed 20 minutes for each actuation in low LCST and high LCST. Blooming by unfolding the petals occurred when the temperature was decreased again in two stages that corresponded to the two different LCST hydrogels.

The flower-shaped multi-transforming actuator was produced by a simple molding technique with UV curing, as illustrated in Fig. 1b. The four-petal flower-shaped mold was made by 3-D printing and the monomer solution was cured in the mold to make the four petals of the flower shape. Two separate four-petal layers were assembled to form the complete flower structure. At first, 5 ml of high LCST (60.8 °C) monomer solution was polymerized in the mold using UV irradiation for 180 s. After removing the gel from the mold, 5 ml of the low LCST (48.0 °C) monomer solution was added to the mold and also polymerized under UV light for 180 s. Each part of the flower was dipped into distilled water for 24 hours to eliminate the unreacted monomer and residues. The two layers were stacked to produce the final 8-petal flower shape and a small amount of f-PNIPAM solution (LCST 48.0 °C) was injected between the centers of the petals. UV irradiation at the center of the flower for 1 minute bonded the two parts together.

The components of every f-PNIPAM are measured by FT-IR spectroscopy (Fig. S2). The results of FT-IR measurement for each f-PNIPAM with different LCST show peaks for their components in similar wavenumber. All of them show peak in 1640 cm^−1^ which means that there are carbonyl groups of acrylamide in f-PNIPAM. They also have peaks of NIPAM where wavenumbers are near 1390 cm^−1^ and 1370 cm^−1^. Peak of HEMA is showed near 1130 cm^−1^ which expresses bond of carbon and oxygen in molecule of HEMA.

This fabrication process generated bending actuation in the f-PNIPAM layers without the use of a passive material to form a bilayer structure. Bending actuation requires a strain mismatch through the film thickness to generate the bending moment. UV curing from one side of the f-PNIPAM monomer solution naturally produced a gradient of polymerization^27^ that also resulted in a variation in water swellability through the film thickness. The intensity of UV light decreases through the monomer solution depth and results in a gradient in the degree of polymerization and crosslinking. More fully polymerized material at the top surface of the film is more densely crosslinked and swells to a lesser degree than the less crosslinked material at the opposite gel surface. Heating through the LCST caused bending away from the gel surface that was closest to the UV source suggesting that the gel volume change at the LCST transition is enhanced in less crosslinked f-PNIPAM gels.

For demonstration purposes, gel sheets were fabricated by equal UV irradiation applied to both sides of the gel sheet. The gel sheets showed the expected change of swelling ratio (Fig. S3) and volume contraction but no bending actuation when heated through the LCST (Fig. S4) because of the symmetry in crosslink density through the film thickness. The difference between crosslinking densities in top and bottom side of f-PNIPAM sheets was observed in measurement of FT-IR spectroscopy. Densely crosslinked polymer chain generally makes large peak at FT-IR spectrum. FT-IR spectrums of top and bottom side of f-PNIPAM hydrogel sheets showed difference in wavenumber near 1170 cm^−1^ which expresses the existence of single bond between carbon molecules. This bond was made up when f-PNIPAM is polymerized under UV light. Every f-PNIPAM with different four LCST showed larger peak in 1170 cm^−1^ of wavenumber when they are measured their top side (Fig. S5). The gel volume of these symmetrically crosslinked sheets was also affected by the UV exposure time with shorter times giving a greater volume reduction during the LCST transition.

The extent of sheet curvature for samples prepared by UV exposure from one side only was also found to depend upon the UV curing time. The curvature when the sheets were heated through the LCST increased with increasing UV irradiation to around 200 seconds (Fig. S6). Longer UV exposure times produced films that displayed smaller curvatures, presumably due to a decrease in crosslink density gradient through the film thickness. UV curing from one side of the film for 200 seconds gives the optimal difference in volume change between the top and bottom surfaces when heated through the LCST.

### Analysis for actuation

**Figure 2:**
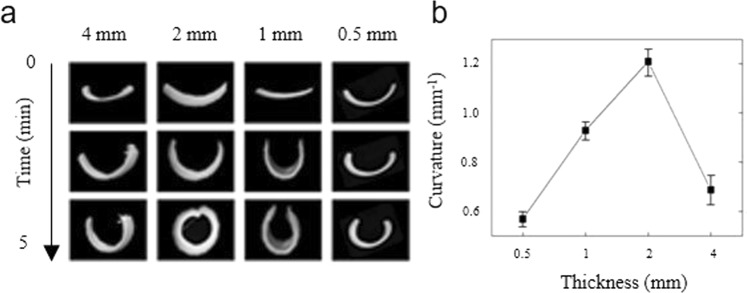
Actuation properties of f-PNIPAM prepared with different length: thickness ratios. The samples through the LCST caused bending away from the top surface. (**a**) Photographs during heating to above the LCST of four f-PNIPAM sheets prepared to the indicated length: thickness ratios. (**b**) Variation in the final curvature of the f-PNIPAM above the LCST as a function of the initial length: thickness ratio. The error bars represent the variation in the degree of curvature along the sample length.

The degree of bending of the f-PNIPAM actuators were also found to be affected by the sheet thickness. Figure 2a shows rectangular shaped hydrogel sheets with different thickness and their bending when the temperature was heated through the LCST. Every polymer sheet was fabricated by UV polymerization of the fPNIPAM solution on a 40 mm length and 10 mm width rectangular mold. The thicknesses were 4 mm, 2 mm, 1 mm, and 0.5 mm. All sheets bent in the same direction near their LCST and the final curvature was determined after the sheet was held for 300 seconds at 50 °C. The sample with a 2 mm thickness showed sufficient curvature to form a complete ring. By contrast, the bands with 0.5 mm, 1 mm, 4 mm thickness generated a U shape at the end of their actuation. The final bending curvatures are represented in Fig. 2b. The graph shows a clear peak and resembles the behavior observed for different curing times. It is concluded that changing the sheet thickness alters the gradient in crosslink density through the sheet thickness and the optimal bending curvature is achieved for 2 mm thick films. Thinner films are likely to have a more consistent crosslink density so that a smaller strain mismatch occurs at the LCST. Thicker films naturally resist bending due to their higher flexural stiffness so that smaller final curvatures are expected.

**Figure 3:**
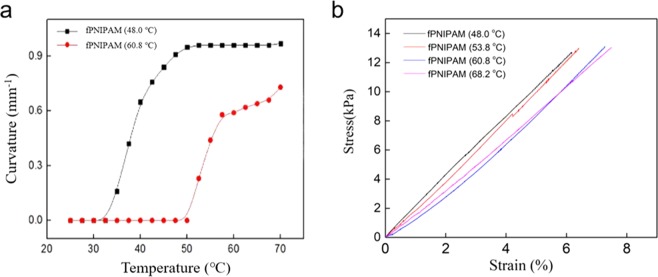
Comparison of bending curvature, LCST and mechanical properties of f-PNIPAM actuators. (**a**) Curvature as a function of temperature with the increase in curvature occurring near LCST; (**b**) Strain-stress curves of sheets fabricated with four f-PNIPAMs with distinctive LCSTs, as indicated.

When the polymer sheets have the same length/thickness ratio, the curvature when heated above LCST also depends on the gel composition. The sheets having a higher LCST show a lower degree of bending curvature. Figure 3a shows the curvature of two different hydrogel films with increasing temperature. Both gels show a near step change in curvature at the LCST. The f-PNIPAM sheet formulated with a LCST near 48.0 °C bends to 0.9 mm^−1^ of degree of curvature. On the other hand, the f-PNIPAM sheet having a higher LCST (60.8 °C) shows the degree of bending curvature of nearly 0.7 mm^−1^.

However, tuning the LCST does not greatly change the mechanical properties of f-PNIPAM sheets (Fig. 3b). The strain–stress curves of four f-PNIPAM sheets with different LCSTs (48.0 °C, 53.8 °C, 60.8 °C, 68.2 °C) were very similar to each other. All f-PNIPAM sheets show a failure strength between 12 kPa and 14 kPa. Strain-to-failure of all fPNIPAM sheets are in the range 5–7%.

### Other application

**Figure 4:**
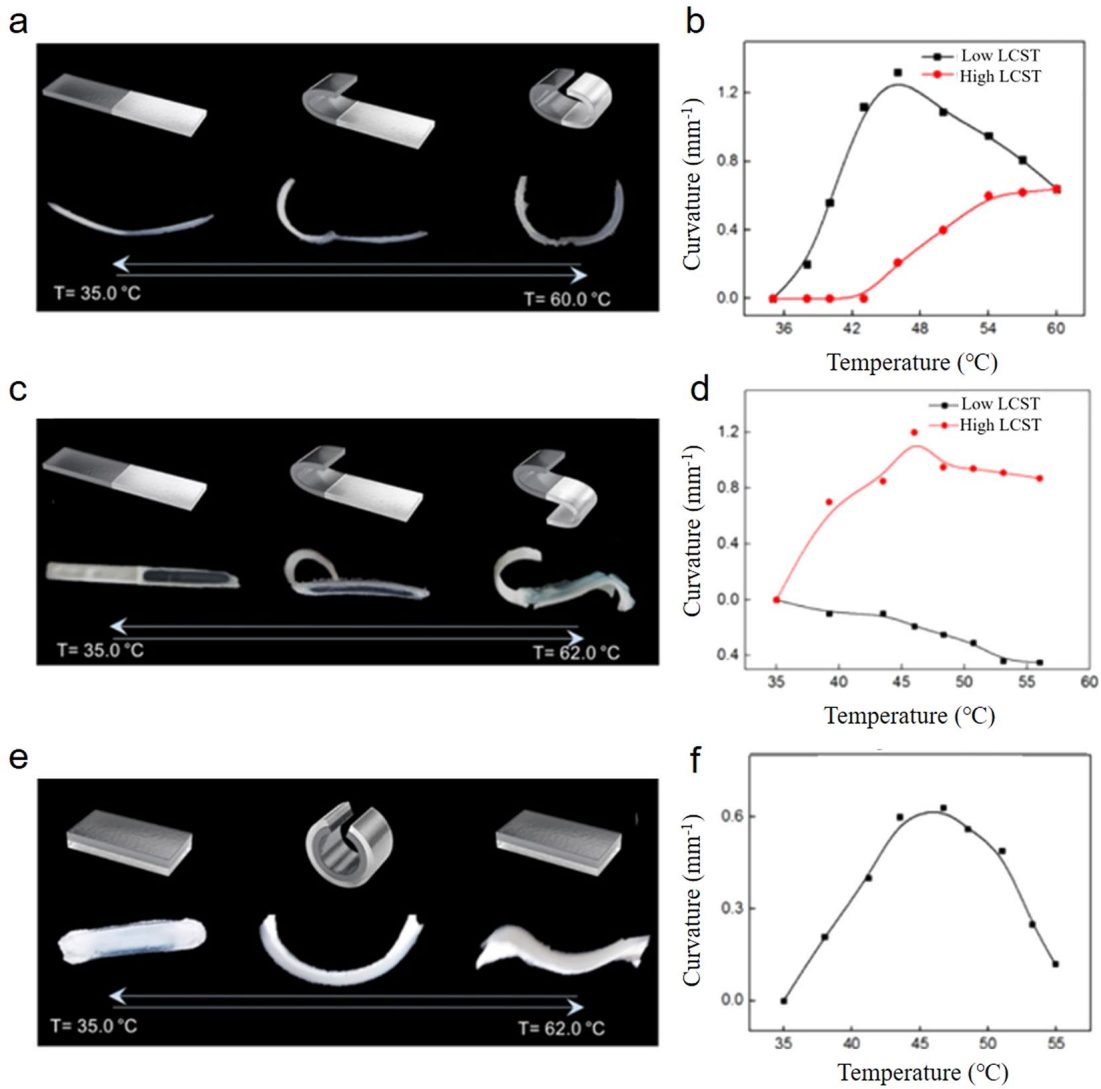
Diverse actuation by different LCST phase transitions. (**a**) The half-to-half structure consists of two different f-PNIPAMs which have low LCST (48.0 °C) and high LCST (60.8 °C). (**b**) Analysis of curvature change in (**a**) with increasing temperature. (**c**) Inverted half-to-half structure consisting of two different f-PNIPAMs with the opposite orientation in each half. One side has low LCST (48.0 °C) and the other side has high LCST (60.8 °C) (**d**) Analysis of curvature change in (**c**) with increasing temperature. (**e**) Structure of layer-on-layer and (**f**) its curvature analysis.

Figure 4 shows the diverse shape morphing abilities of the multi-transforming actuated devices. With different LCST f-PNIPAM solutions, various basic configurations were demonstrated such as half-to-half, inverted half-to-half and layer-on-layer structures. The half-to-half structure consists of two f-PNIPAM sheets connected in series (Fig. 4a). One half consists of a 40 mm long low LCST (48.0 °C) f-PNIPAM sheet and the other half consists of a high LCST (60.8 °C) f-PNIPAM sheet. The whole hydrogel sheet is 2 mm thick. When heated from ambient temperature, the low LCST part starts to bend near 35 °C and it finishes its bending actuation near 50 °C. The curvature degree of the low LCST part shows a peak above 1.2 mm^−1^ near 45 °C and decreases after the peak (Fig. 4b). With the high LCST part, the curvature degree shows a change when the temperature rises from 50 °C to 60 °C reaching a final curvature of 0.8 mm^−1^. Actuation images of the half-to-half structure during heating are illustrated in Fig. 4a. The inset images show schematically the transitions for both parts of the half-to-half structure. Each parts of half-to-half structure took 10 minutes for their actuation.

Figure 4c shows the actuation of an inverted half-to-half structure. The orientations of the two halves of this actuator were inverted so that the sheets bend in different directions. Again the two parts show distinctive bending behavior as the temperature rises. The sheets transform to an S shape as the result of multi-transformation. In Fig. 4b and d, the degrees of curvature are decreased at temperatures after the peak. This peak incurvature was also observed in a single sheet of PNIPMA actuator with a 48 °C LCST (Fig. S7). The small loss in curvature at temperatures above LCST may be associated with network rearrangement or relaxation.

The layer-on-layer device shows a bending and straightening transformation when heated (Fig. 4e,f). The upper layer consists of a low LCST hydrogel sheet and the bottom layer consists of a high LCST hydrogel sheet. These two layers contract near their LCSTs, and thus show bending and unbending while heating and with no cooling. Near the lower LCST, only the upper layer shrinks first and the sheet curves upward. When the temperature rises near the higher LCST, the upper layer finishes its response and the lower layer starts shrinking. As a result of this shrinking, the structure unbends and goes back to a near flat state. Each phase of layer-on-layer actuation needed 10 minutes to complete bending and unbending. Figure 4e shows the schematic illustration and optical images of the transformation of the layer-on-layer structure and its actuation.

## Discussion

In this research, we reported multi-transform artificial muscle actuated structures by using multiple actuators with distinct transformation stimuli and simple moulding to fabricate shapes. NIPAM, HEMA, and AM monomer were used to control the LCSTs. Furthermore, it was found that the UV curing of the monomer mixture produced sheets with a gradient in crosslink density so that the sheets bent during heating through the LCST. The degree of bending depended on the polymer composition, the crosslink density gradient and the film thicknesses. Devices made using these actuators has the advantage of being able to show multi-transformations with simple modifications to the structural design. Therefore, this study is expected to be applied to soft and smooth biomimetic motion and soft robot research.

## Method

### Preparing a functionalized PNIPAM (f-PNIPAM) solution

PNIPAM, acrylamide (AM), 2,2′-hydroxyethyl methacrylate (HEMA), *N*,*N*′-methylenebisacrylamide (BIS), and IRGACURE 2959 were purchased from Sigma-Aldrich (USA). A solution was prepared using deionized water from a Milli-Q plus water purification system (Millipore, Bedford, UK). To make the first f-PNIPAM solution that has 48 °C LCST, 2.04 g of PNIPAM was dissolved in 10 ml of distilled water. Then, after the PNIPAM was completely dissolved, 0.412 g of AM and 0.031 ml of HEMA were added to the PNIPAM solution. After that, the three mixed monomers were vortexed until completely dissolved. For polymerization, 0.102 g of BIS and 0.01 g of IRGACURE 2959 were added. To make the second f-PNIPAM solution, which has 60 °C LCST, 1.856 g of PNIPAM, 0.61 g of AM, 0.033 ml of HEMA, 0.0928 g of BIS, and 0.01 g of IRGACURE 2959 were used. They were made in the same way as the first f-PNIPAM solution. All f-PNIPAM solutions are referenced to the amounts in Table 1.

**Table 1:** Different LCSTs by components of NIPAM, HEMA, and AM monomers.

	PNIPAM	AA	HEMA	LCST
1	8.160 g	1.648 g	0.1256 ml	48.0 °C
2	7.816 g	2.043 g	0.1280 ml	53.8 °C
3	7.424 g	2.440 g	0.1312 ml	60.8 °C
4	7.0 g	2.856 g	0.1336 ml	68.2 °C

### UV polymerization condition

To UV polymerize the prepared f-PNIPAM solution, a UV source (Dymax, Bluewave200, USA) was used. Wavelength and intensity of UV light were 375 nm and 17 W/cm^2^, respectively, and the length between the light source and monomer solution was 15 cm. Every actuator was polymerized in this same condition, and the thickness of all actuators was 2 mm, unless otherwise indicated.

### Making a flower-shaped actuator

To make the flower-shaped actuator, we used a 3-D printed polytetrafluoroethylene (PTFE) mold using a Fused Deposition Modeling (FDM) 3-D printer (Ultimaker2+, Netherlands). After that, according to the composition ratio of NIPAM, HEMA, and AM monomer, an aqueous solution of f-PNIPAM having four different LCSTs was injected into the PTFE mold. The size of the mold was larger than that of the UV light source region and could not be cured at one time; therefore, the half part of each layer was radiated in curing. UV light was shone for 180 s. After polymerization, the whole flower-shaped actuator was detached from the PTFE mold. It was dipped into deionized water for 24 hours for fully swelling and washing.

### Making method for other different LCST actuators

The rectangular mold was also made using the FDM 3-D printer using PTFE. The rectangular mold size was 1 cm × 8 cm. Half-to-Half structures were made in such a way that one LCST polymer was made first, followed by the remaining second LCST polymer. The two polymers were connected as one polymer during UV curing due to BIS. Each different LCST film-type polymer size was 1 cm × 4 cm. In the case of 4 cm sheets, the curing time was 180 s. To make a layer-on-layer structure, it was completed by placing another LCST polymer on the underlying LCST polymer. Each LCST film-type polymer was cured for 180 s. The whole layer-on-layer actuator size was 1 cm × 4 cm. All actuators are dipped into unionized water for washing and swelling. They stayed in deionized water for 24 hours.

### Actuation test and analysis

Every actuation was observed in water, and a video was taken using a custom-made USB camera. The degree of curvature was measured by an image capture program (1 frame/s using ImageJ image-processing software; http://rsb.info.nih.gov/ij/). For the deformation of the flower-shaped actuator, the water was heated from 35 °C to 65 °C for 1 hour. The temperature was maintained at 50 °C for 20 minutes. The Half-to-Half structure showed that the first deformation started near 48 °C and the actuation ended after 10 min. After half of the sheet transformed, the water was heated to 60 °C for the second deformation. The second actuation started at  60.8 °C and the end of the actuation was at 62 °C. For the layer-on-layer structure actuation test, the temperature was controlled in the same way as the Half-to-Half structure. It took 1 hours for each actuation test for these two kinds of structures. For the test in Fig. 2, we put the sheets in water at 50 °C for 10 minutes and observed the actuation.

In experiment of Fig. 3a, the f-PNIPAM hydrogel sheets putted in the water at 24 °C. The water is heated to 70 °C for 30 minutes.

### Modulus test

The mechanical properties were measured using a UTM (Shimadzu model: EZ-SX, Japan) at a loading rate of 50 μm min^−1^. The mechanical properties of the total hydrogel sheets were calculated by integrating stress–strain curves, which were measured using a microbalance (Mettler Toledo, XP2U Balance, USA).

## Supplementary information

Supporting information
