# Supplementary material for: Biomimetic Thermal-sensitive Multi-transform Actuator
Source: Sci Rep. 2019 May 27;9:7905. doi: 10.1038/s41598-019-44394-x (PMC6536525; doi:10.1038/s41598-019-44394-x)
Supplement: Supplementary file 1 — Supporting information [file 41598_2019_44394_MOESM1_ESM.docx]

**Biomimetic Thermal-sensitive Multi-transform Actuator**

Tae Hyeob Kim,**^†^** Jung Gi Choi,**^†^** Ju Hyeong Byun, Yongwoo Jang, Sung Min Kim, Geoffrey. M. Spinks and Seon Jeong Kim*

[a] T. H. Kim, J. G Choi, J. H Byun, Prof. Y. Jang, Prof. S. J Kim*

Center for Bio-Artiﬁcial Muscle, Department of Biomedical Engineering, Hanyang University, Seoul 04763, South Korea

[b] Prof. S. M. Kim

Department of Physical Education

Hanyang University, Seoul 04763, South Korea

[c] Prof. G. M. Spinks

ARC Centre of Excellence for Electromaterials Science, Intelligent Polymer Research Institute, University of Wollongong, Wollongong, New South Wales 2522, Australia.

*****To whom correspondence should be addressed: [sjk@hanyang.ac.kr](mailto:sjk@hanyang.ac.kr)

**^†^** These authors contributed equally to this work.


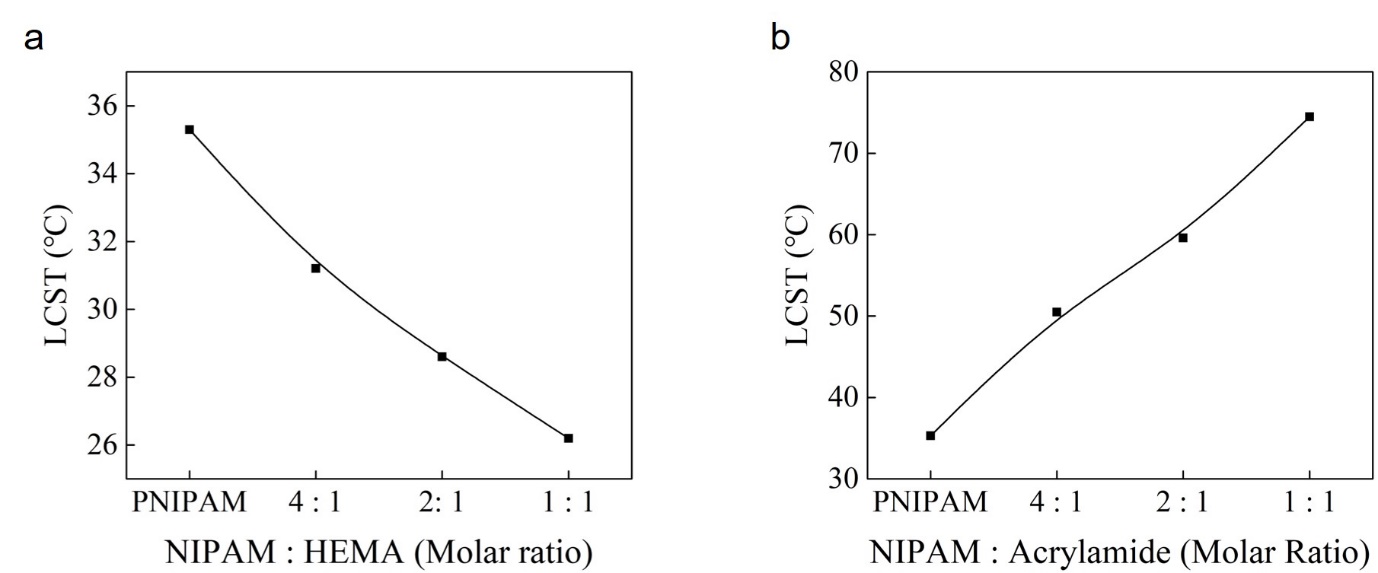


**Figure S1** LCST shifts with PNIPAM hydrogel containing (a)HEMA, and hydrogel containing (b)acrylamide.

**
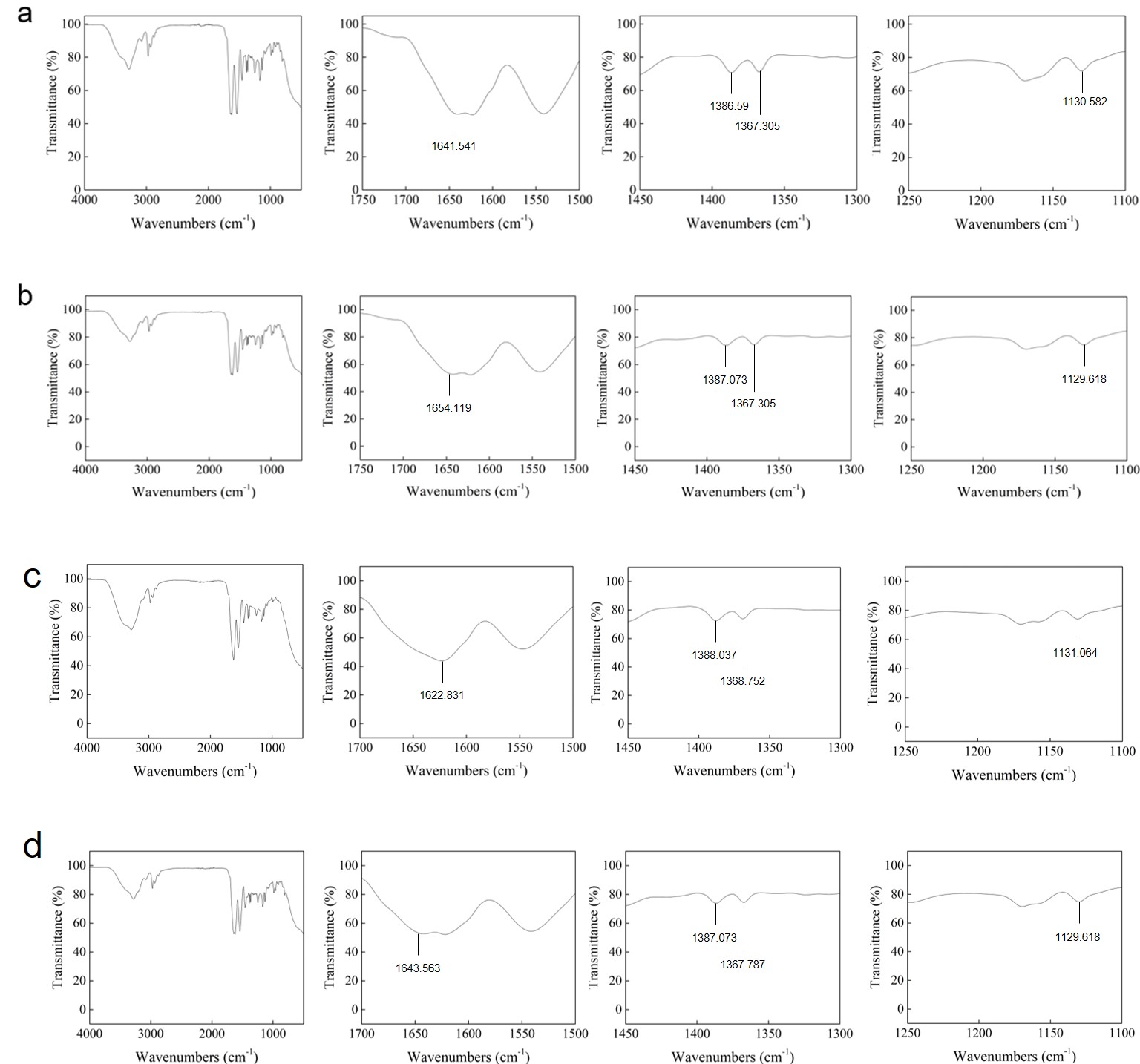
**

**Figure S2.** The FT-IR spectrum of four kind of f-PNIAM with different LCST. a) FT-IR spectrum of f-PNIPAM(48℃). b) 53.8℃. c) 60.8℃. d) 68.2℃. The peak near 1640 shows carbonyl groups of acrylamide are in f-PNIPAM polymer. The spectrums show two peaks between 1350 and 1400 of NIPAM. They also show the peak of HEMA near 1130.

**
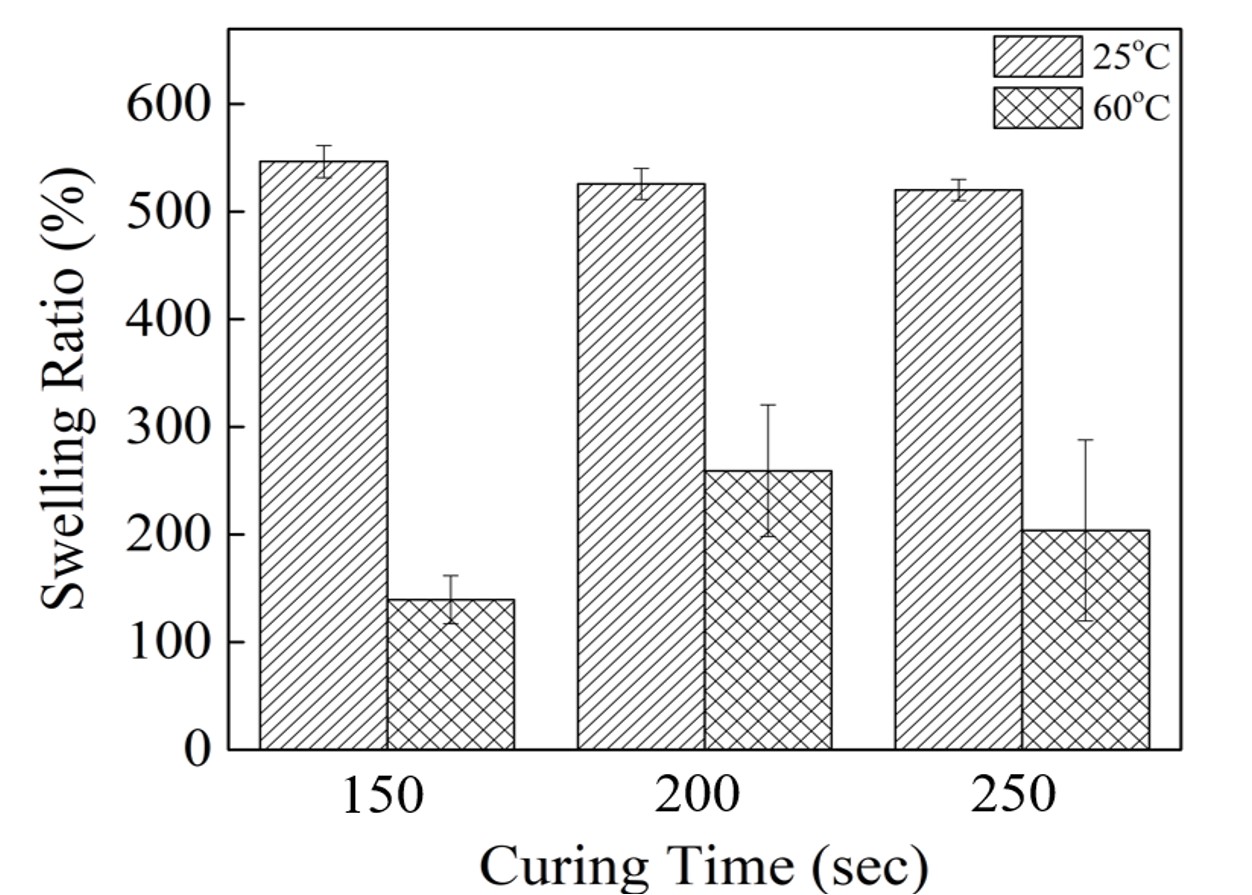
**

**Figure S3.** Equilibrium swelling ratio in water at 25°C and 60°C of sheets cured by exposing both sides to UV irradiation for different curing times.

**
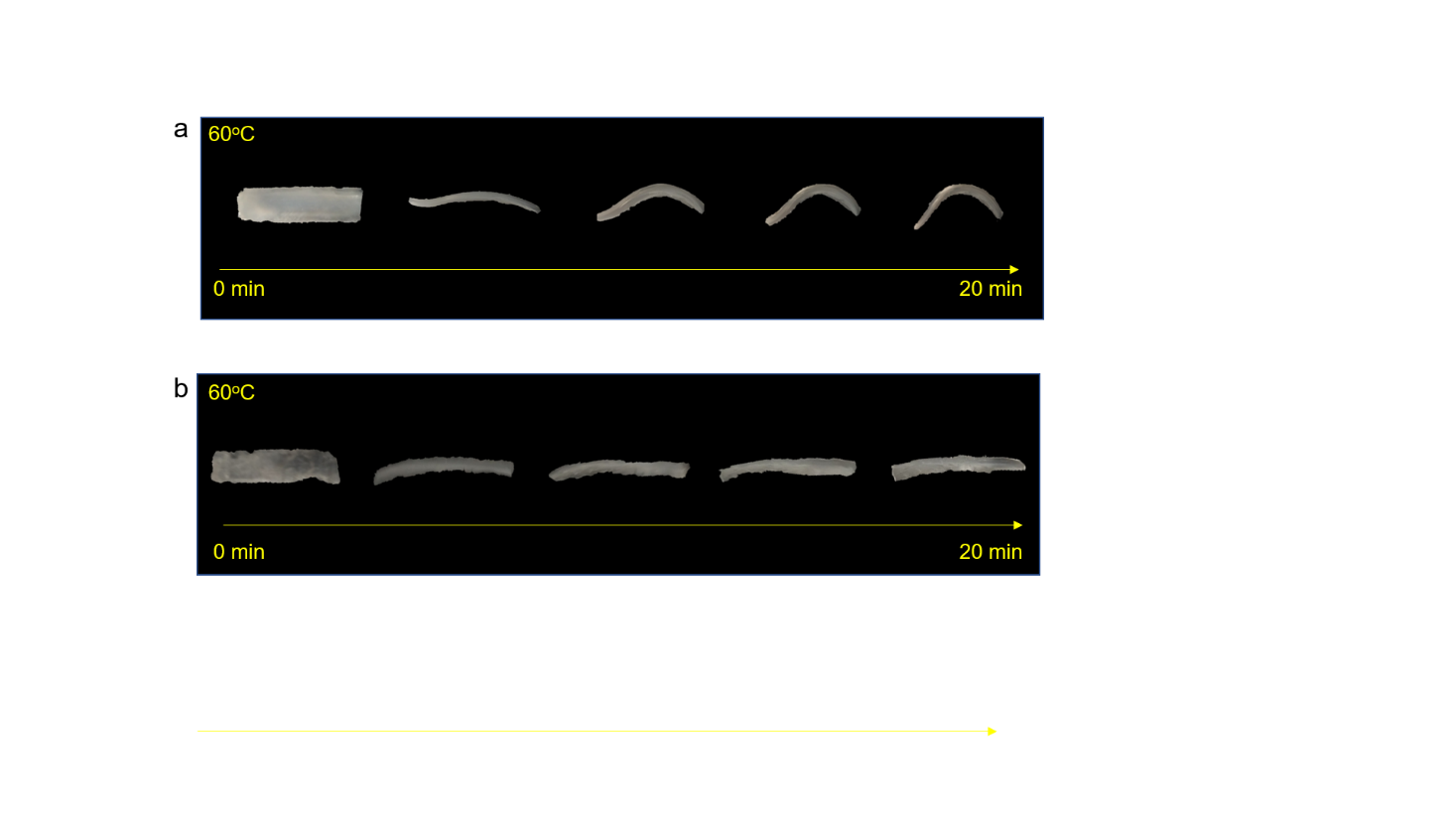
**

**Figure S4.** Comparison of motion performance between both sides cured sheet (b) and one side cured sheet (a).

**
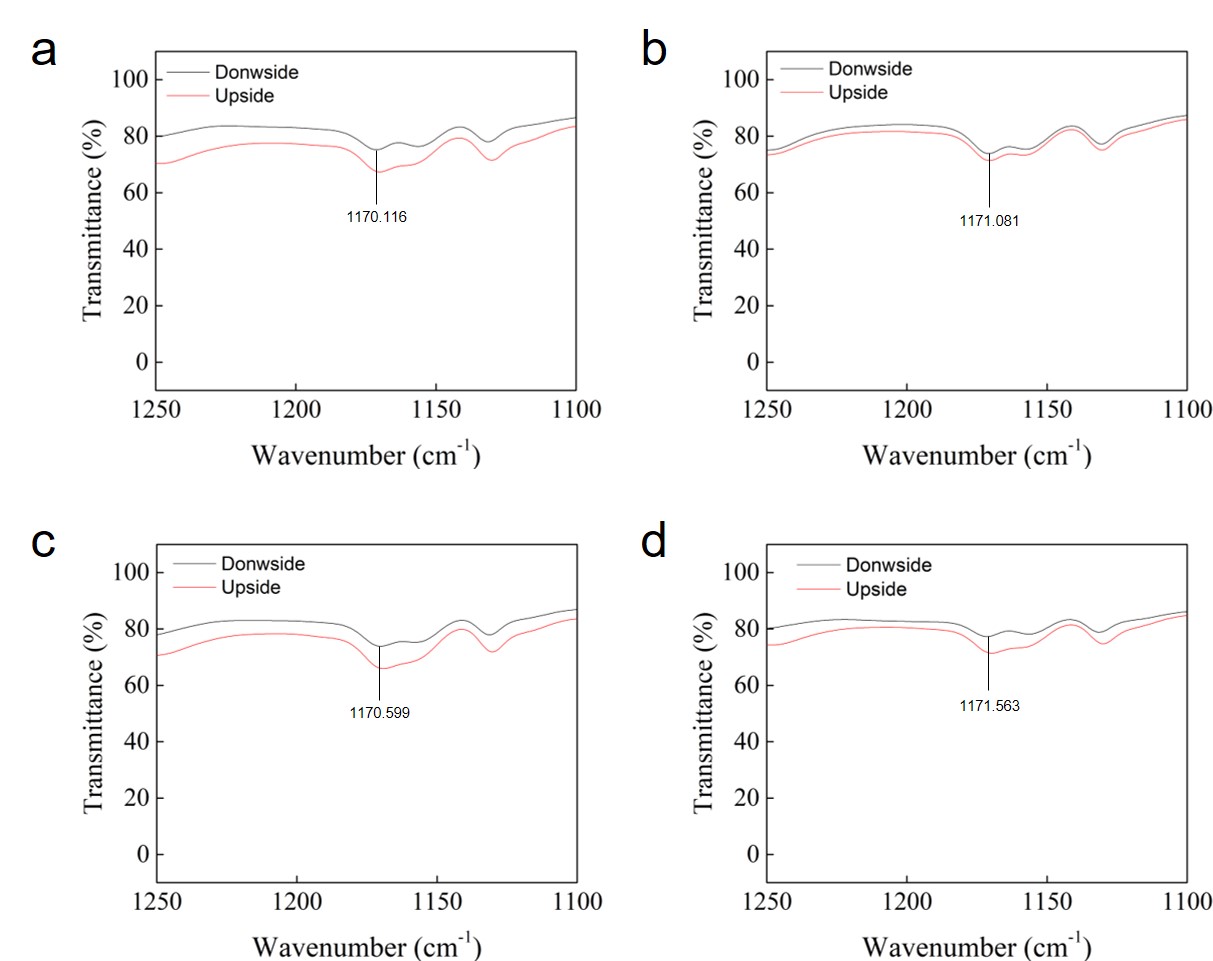
**

**Figure S5** Results of FT-IR spectroscopy about top and bottom side of f-PNIPAM with different LCST. a) Spectrum of f-PNIPAM(48℃). b) 53.8℃. c) 60.8℃. d) 68.2℃. Peak near 1170 means the single bonds between carbon molecules which are made up during polymerization of f-PNIPAM.

**
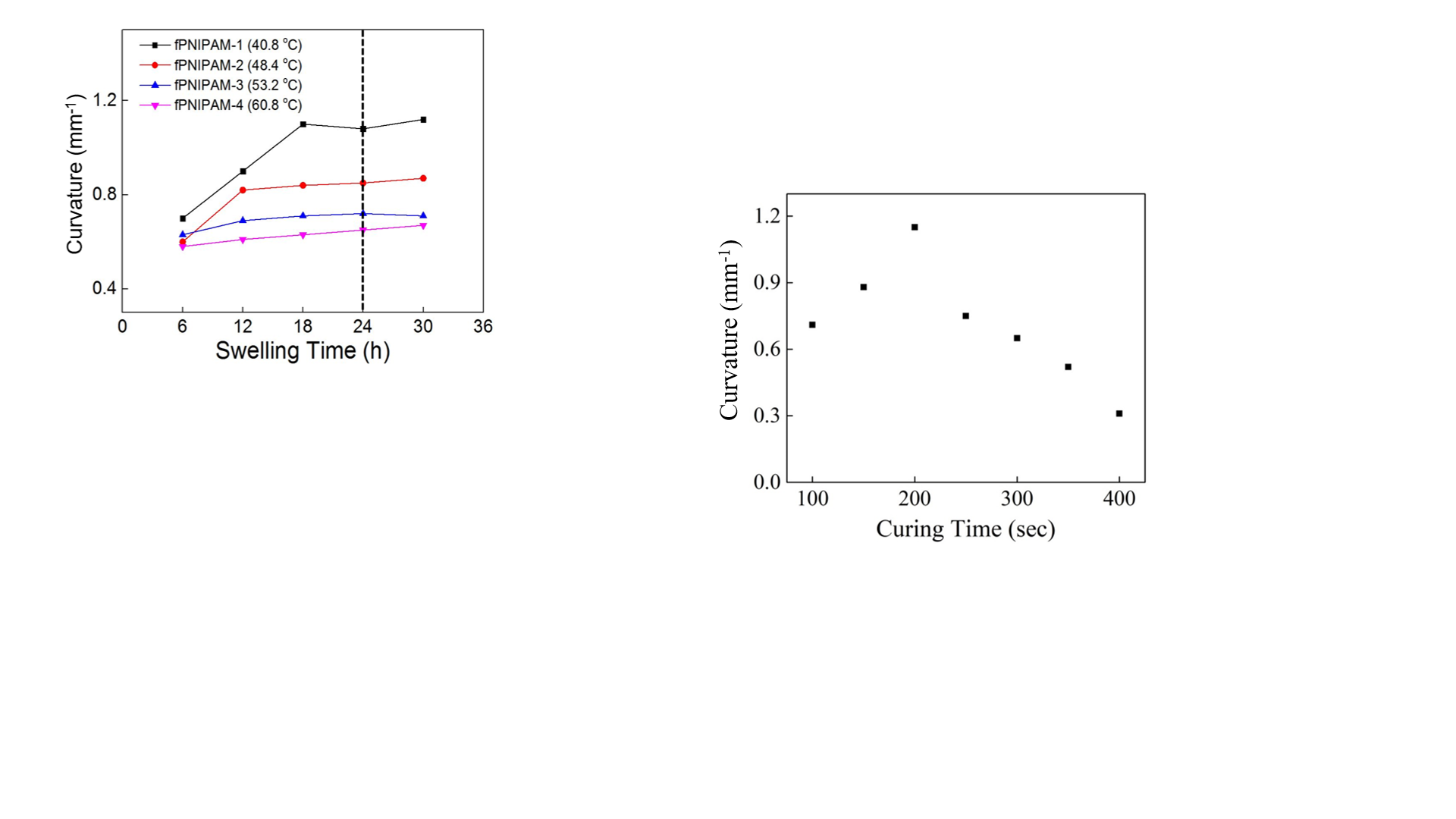
**

**Figure S6.** Comparison of final curvature above LCST for samples prepared with different UV-curing times.


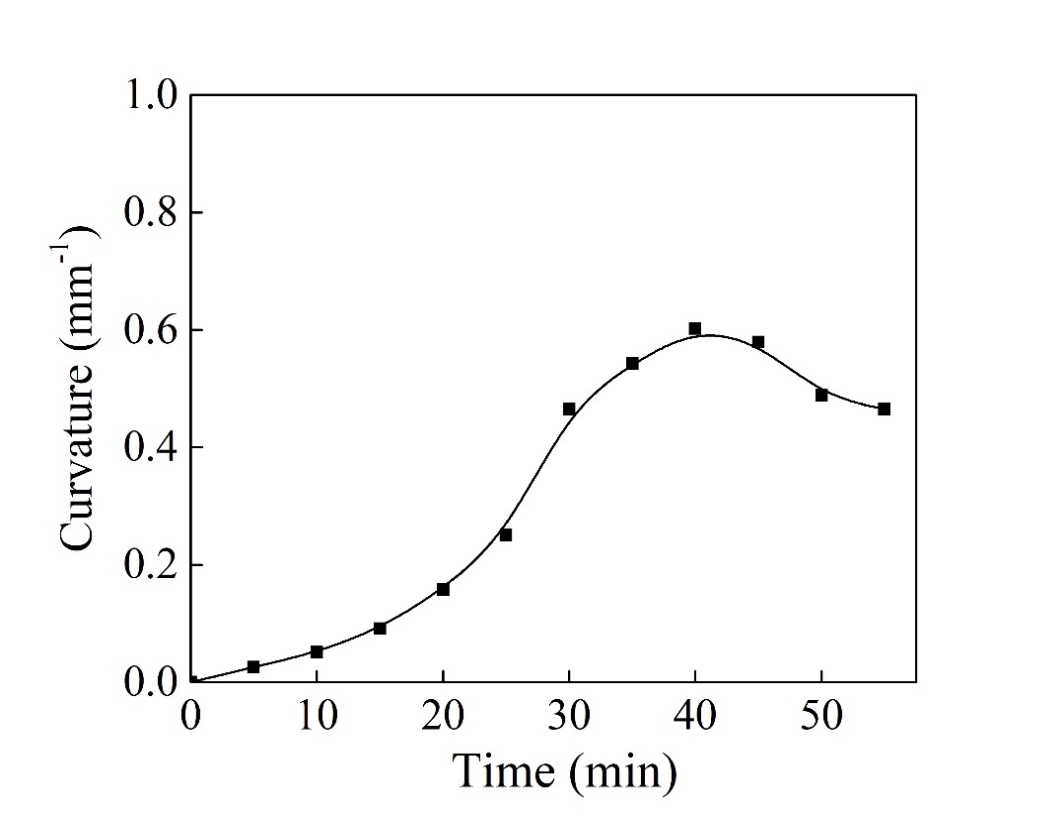


**Figure S7.** Curvature change with time. PNIPAM sheet which has 48 ^o^C of LCST is observed its curvature in water heated from 54 ^o^C to 67 ^o^C for 55 minutes.


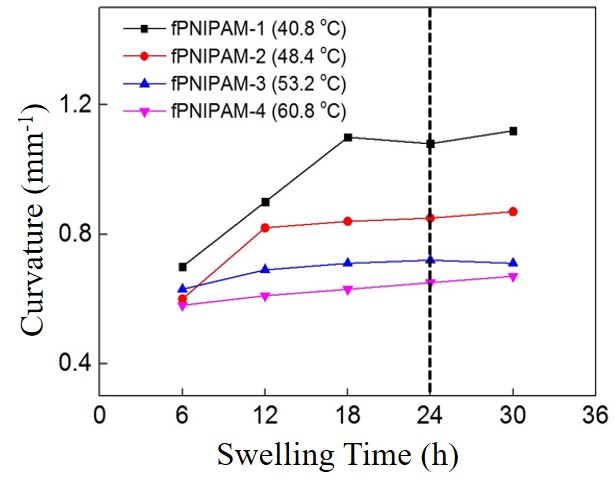


**Figure S8.** Actuation performance of each f-PNIPAM by water swelling time.
